# Development of Graphene Oxide-Based Anticancer Drug Combination Functionalized with Folic Acid as Nanocarrier for Targeted Delivery of Methotrexate

**DOI:** 10.3390/pharmaceutics16060837

**Published:** 2024-06-20

**Authors:** Reyhan Yanikoglu, Canan Yagmur Karakas, Fatih Ciftci, Mert Akın Insel, Zeynep Karavelioglu, Rahmetullah Varol, Abdurrahim Yilmaz, Rabia Cakir, Hüseyin Uvet, Cem Bulent Ustundag

**Affiliations:** 1 Department of Bioengineering, Faculty of Chemical and Metallurgical Engineering, Yıldız Technical University, İstanbul 34210, Türkiye; 2 Department of Food Engineering, Faculty of Chemical and Metallurgical Engineering, Yıldız Technical University, İstanbul 34210, Türkiye; 3 Department of Biomedical Engineering, Fatih Sultan Mehmet Vakif University, Istanbul 34445, Türkiye; 4 Department of Chemical Engineering, Faculty of Chemical and Metallurgical Engineering, Yıldız Technical University, İstanbul 34210, Türkiye; 5 Department of Health Sciences and Technology, ETH Zurich, 8092 Zurich, Switzerland; 6 Department of Mechatronics Engineering, Yildiz Technical University, Istanbul 34349, Türkiye; 7 Business Administration Department, Bundeswehr University Munich, 85579 Munich, Germany; 8 Türkiye Biotechnology Institute, Health Institutes of Türkiye, Istanbul 34718, Türkiye; 9 Health Biotechnology Joint Research and Application Center of Excellence, Istanbul 34220, Türkiye

**Keywords:** carbon materials, cancer therapy, drug delivery, methotrexate, folic acid

## Abstract

Graphene has become a prominent material in cancer research in recent years. Graphene and its derivatives also attract attention as carriers in drug delivery systems. In this study, we designed a graphene oxide (GO)-based methotrexate (MTX)-loaded and folic acid (FA)-linked drug delivery system. MTX and FA were bound to GO synthesized from graphite. MTX/FA/GO drug delivery system and system components were characterized using Fourier transform infrared spectroscopy (FTIR), differential calorimetric analysis (DSC), scanning electron microscopy (SEM), transmission electron microscopy (TEM), zeta potential analysis, and dimension measurement (DLS) studies. SEM and TEM images confirmed the nanosheet structure of GO synthesized from graphite, and it was shown that MTX/FA binding to GO transformed the two-dimensional GO into a three-dimensional structure. FTIR and DSC graphs confirmed that oxygen atoms were bound to GO with the formation of carboxylic, hydroxyl, epoxide, and carbonyl groups as a result of the oxidation of graphite, and GO was successfully synthesized. Additionally, these analyses showed that MTX and FA bind physicochemically to the structure of GO. The in vitro Franz diffusion test was performed as a release kinetic test. The release kinetics mathematical model and correlation coefficient (R2) of MTX-loaded GO/FA nanomaterials were found to be the Higuchi model and 0.9785, respectively. Stiffness analyses showed that adding FA to this release system facilitated the entry of the drug into the cell by directing the system to target cells. As a result of the stiffness analyses, the stiffness values of the control cell group, free MTX, and MTX/FA/GO applied cells were measured as 2.34 kPa, 1.87 kPa, and 1.56 kPa, respectively. According to these results, it was seen that MTX/FA/GO weakened the cancer cells. Combined use of the MTX/FA/GO drug delivery system had a higher cytotoxic effect than free MTX on the MDA-MB-231 breast cancer cell line. The results showed that the synthesized MTX/FA/GO material has promising potential in cancer cell-specific targeted therapy for MTX as a drug delivery system.

## 1. Introduction

Cancer is a pervasive and serious health issue that continues to experience a growing incidence among humans in contemporary society [1]. Despite advancements in treatment, it still frequently leads to fatalities [2]. Cancer encompasses a diverse range of diseases that can manifest in various tissues throughout the body and usually starts with the uncontrolled proliferation of a cell in the tissue and spreads to other tissues [3]. The most common cancer cases today are breast, lung, colon, rectum, and prostate cancers. Breast cancer is a type of cancer that can occur in both men and women, but is much more common in women, and can have different types [4]. In their quest to find innovative approaches to combating cancer, researchers have explored the potential to use anticancer drugs more effectively with targeted systems.

GO, a derivative of graphene, has garnered attention for its unique properties that could be harnessed in cancer therapy. GO has a large two-dimensional surface area and allows for efficient loading of drug molecules, and thanks to its nano-sized structure, it can interact with cells more effectively and aid in targeted drug delivery [5]. This has led researchers to use GO to deliver anticancer drugs directly to tumor sites. However, it is important to note that although GO is promising in research settings, its clinical application as a cancer drug carrier is still an ongoing area of research, and there are no specific drug formulations or treatments for cancer that include GO [6,7,8].

FA, also known as vitamin B9, has been investigated for its potential in cancer therapy, particularly in the context of targeted drug delivery [9,10]. Many cancer cells overexpress folate receptors on their surfaces, which allows them to take up more folic acid than normal cells. This characteristic has led to the development of FA-based targeting strategies in cancer therapy. FA can be conjugated to drugs, nanomaterials, or other therapeutic agents. These agents are designed to specifically deliver treatment to cancer cells that overexpress folate receptors. The idea is that cancer cells would take up more of the drug-loaded folic acid complexes compared to healthy cells, leading to a more targeted and potentially effective treatment. This approach aims to reduce side effects by minimizing damage to normal cells. Nanomaterials functionalized with FA can be used to deliver therapeutic agents directly to cancer cells [11,12,13,14]. These nanomaterials can carry drugs, genes, or other treatment components. FA on the surface of nanomaterials enables them to bind to cancer cells, enhancing selective delivery. FA can be combined with imaging agents to aid in cancer detection and monitoring. By conjugating FA with imaging probes, it becomes possible to identify and visualize tumors that have elevated folate receptor expression. While this is not directly related to using FA itself as a treatment, certain cancer therapies involve targeting folate metabolism [15,16,17,18,19]. FA binds to FA receptors present on the surface of breast cancer, which are usually highly expressed. This ensures that the drug delivery system produced is targeted [20,21,22].

MTX is an anticancer drug that interferes with the growth of cancer cells by inhibiting a key enzyme involved in DNA synthesis [23,24,25,26,27]. While MTX can be effective in treating certain types of cancer, its use can also lead to side effects and limited targeting of cancer cells. Drug delivery strategies involving carriers such as nanomaterials can enhance the effectiveness and specificity of MTX treatment. Nanomaterials are tiny particles on the nanometer scale that can be engineered to carry drugs [28,29]. MTX can be encapsulated within nanomaterials, which serve as carriers. These nanomaterials can improve the drug’s solubility, stability, and bioavailability. Additionally, they can be functionalized with targeting agents, such as antibodies or ligands, to enhance specificity for cancer cells. Functionalized nanomaterials carrying MTX can specifically target cancer cells that express certain surface receptors. This targeted approach increases the drug’s accumulation in cancer cells while minimizing exposure to healthy cells, potentially reducing side effects. Nanomaterials can also be designed for the sustained release of MTX over time. This controlled release can lead to prolonged therapeutic levels of the drug, reducing the need for frequent dosing. Nanomaterials can carry multiple therapeutic agents, allowing for combination therapies. MTX-loaded nanomaterials can be combined with other drugs to create synergistic effects and improve treatment outcomes [30,31,32,33].

In this study, the purpose of the carrier nanosystem (GO) is to carry the therapeutic agent (MTX) and targeting agent (FA) to facilitate targeted drug delivery to cancer cells. This strategy aims to increase the drug’s effectiveness while minimizing its impact on healthy tissues, thus reducing potentially negative side effects. There have been some studies on MTX and/or folic acid loaded on GO [23], gold nanoparticles [25], and dendrimer nanoparticles [32]. However, to the best of the authors’ knowledge, the release kinetics of the MTX/FA/GO combination by the Franz diffusion method and its cytotoxic effect on the MDA-MB-231 cancer cell line have not been investigated. Additionally, the weakening effect of MTX on cancer cells using this combination and its biomechanical effect via stiffness testing have not yet been studied. For this purpose, the cytotoxic effect of MTX on breast cancer MDA-MB-231 cells was investigated using MTX-loaded and FA-functionalized GO as a pharmaceutical agent compared to MTX alone.

## 2. Materials and Methods

### 2.1. Materials

In this study, the chemicals used for synthesis, such as graphene flake (mesh size 300), sulfuric acid (H_2_SO_4_), potassium permanganate (KMnO_4_, 99.9%), phosphoric acid (H_3_PO_4_), methotrexate, hydrogen peroxide (H_2_O_2_, 30%), NaNO_3_, and ethanol, were purchased from Merck and Sigma Aldrich (St. Louis, MO, USA). Folic acid was obtained from Ruber Biotechnology (Ankara, Türkiye). The MDA-MB-231 cell line was supplied by American Type Culture Collection (ATCC). Fetal bovine serum (FBS), phosphate-buffered saline (PBS), Dulbecco’s modified Eagle medium/nutrient mixture F-12 (DMEM/F-12), penicillin-streptomycin, trypsin-EDTA, and trypan blue were purchased from Gibco (New York, NY, USA). XTT (2,3-bis-(2-methoxy-4-nitro-5-sulfophenyl)-2H-tetrazolium-5-carboxanilide and phenazine methosulfate (PMS) were obtained from Santa Cruz Biotechnology (Dallas, TX, USA).

### 2.2. Synthesis of Graphene Oxide (GO)

Briefly, 2 g of graphite and 1 g of NaNO_3_ were put into a flask at 0 °C. Then, 50 mL of concentrated H_2_SO_4_ was added to the mixture, which was stirred for 30 min at 5 °C. Subsequently, 7 g of KMnO_4_ was added to the reaction system stepwise over 1 h, while the temperature of the mixture was kept below 20 °C. Then, the temperature was raised to 35 °C and the mixture stirred for 2 h. Afterwards, 90 mL of deionized distilled (DD) water was slowly added to the solution, and the temperature of the reaction system jumped to 70 °C instantly. Finally, 7 mL of H_2_O_2_ (30%) and 55 mL of DD water were poured into the reaction system, resulting in the formation of a bright yellow suspension. The GO was separated by filtration, washed three times with diluted HCl (3%), and then dispersed in DD water. Exfoliation of GO was carried out by sonicating (200 W) the GO in DD water at room temperature for 1 h, generating homogeneous GO dispersions [34].

### 2.3. Drug Loading (MTX) on GO and Conjugation of Folic Acid (FA)

**Figure 1:**
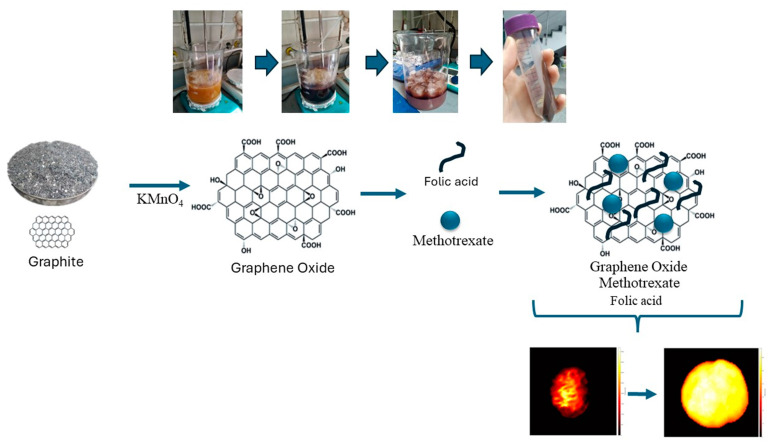
Schematic presentation of fabrication of GO-based MTX delivery system through attachment of FA to the formulations and effects on MDA-MB-231 breast cancer cells. GO: graphene oxide; MTX: methotrexate; FA: folic acid.

The anticancer drug was loaded by adding 1.0 mL of methotrexate (100 µg/mL) to 4.0 mL of GO in buffer solution (25 µg/mL) while stirring for 48 h in the dark at room temperature [23]. FA molecules were conjugated to GO according to the literature [35]. Then, 0.08 g FA was added to the prepared GO, and 12 mL of water was added to the mixture. The mixture was left to stir in the dark for 24 h for binding (Figure 1). After 24 h, the mixture was precipitated by centrifugation (25 °C, 10,000 rpm, 20 min) to separate free MTX and FA in the solution from the MTX/FA/GO system, and the supernatant was separated.

An in vitro release kinetics study was carried out for MTX/FA/GO nanomaterials. Here, the release kinetics efficiency of MTX was observed and compared. Furthermore, kinetic models were obtained for the MTX/FA/GO system and the performance of these models was assessed.

### 2.4. Characterization of GO and Drug Delivery System

#### 2.4.1. Particle Size and Zeta Potential Analysis

The average particle diameter (z average), and zeta potential of the GO and MTX/FA/GO were measured using dynamic light scattering on a Zetasizer Nano ZS (Malvern Instruments Ltd., Malvern, Worcestershire, UK). All samples were diluted 100-fold (*v*/*v*), and measurements were made on freshly prepared GO and MTX/FA/GO three times per sample.

#### 2.4.2. Morphology of the GO and Drug Delivery System

##### Scanning Electron Microscopy (SEM) Imaging

GO and MTX-GO-FA surface morphology was examined using a Quanta 450 FEG SEM (FEI, Graz, Austria). Under vacuum, a 40 mA current density was used to spray-coat each sample with a thin layer of gold-platinum (Agar Manual Sputter Coater B7340; Agar Scientific, Stansted, UK). Under low vacuum conditions, with an acceleration voltage of 7 kV, the samples’ morphology was examined at different magnifications.

##### Transmission Electron Microscopy (TEM) Imaging

Solutions of GO and MTX-FA-GO were applied to 200 mesh carbon-coated copper grids, and the mixture was allowed to cure at room temperature. Subsequently, the grid was dyed using an aqueous solution containing 2% uranyl acetate and allowed to air-dry. The TEM (Hitachi HT7800, Tokyo, Japan) equipped with a digital camera and operated at 100 kV acceleration voltage captured pictures of the GO and MTX-GO-FA structures.

#### 2.4.3. Fourier Transform Infrared Spectroscopy (FTIR)

An FTIR spectrophotometer (ATR-FTIR; Bruker Tensor 27, Karlsruhe, Germany) was used to acquire all samples’ Fourier transform infrared spectra. At a wavelength range of 4000–500 cm^−1^, all spectra were recorded in absorption mode with a resolution of 4 cm^−1^.

#### 2.4.4. Differential Scanning Calorimetric Analysis (DSC)

Under nitrogen gas flow, the thermal characteristics of the samples were measured using DSC (SDT Q600, TA Instruments, New Castle, DE, USA). Five to eight milligram samples were precisely weighed, put into aluminum vials, and sealed tightly. The sealed samples were heated between 20 and 250 °C at 10 °C/min while 20 mL/min of nitrogen flow was applied.

### 2.5. In Vitro Study: Franz Diffusion Drug Delivery Kinetics

The most common in vitro technique for measuring dermal absorption is to apply an active ingredient in an appropriate formulation to the surface of a skin sample [36,37]. The most commonly used dissolution test methods for MTX/FA/GO were performed using the Franz diffusion device [38,39]. An active MTX/FA/GO sample was placed on the membranes with Franz diffusion cells. The membrane is located in the middle of the Franz device. The MTX/FA/GO sample was placed on the membrane. A medium (2.5 mL) was added to the lower part of the cells, and the sample (1.5 mL) was examined through the upper part of the cells. The ambient temperature was kept constant at 37 °C, and the experiment was carried out with constant speed mixing. At the 15th and 30th min during the 1st, 2nd, 4th, 6th, 8th, 24th, 30th, 36th, 42th, and 48th h, 2.5 mL of the sample was taken from the lower part and replaced with a fresh medium brought to 37 °C. The experiment was continued. The amount of active substance in the samples was determined using HPLC. The % cumulative amount of the active substance passing through the cells was plotted against time, and the % cumulative amount passed at the end of 24 h was calculated. Cumulative mass losses for MTX/FA/GO were statistically analyzed using the Minitab 19 program. The encapsulation efficiency (EE%) and MTX (methotrexate) drug loading were calculated using the following equation:

(1) $$\mathrm{EE}\%=\frac{\left(\mathrm{Total}\;\mathrm{concentration}\;\mathrm{of}\;\mathrm{MTX}-\mathrm{Total}\;\mathrm{concentration}\;\mathrm{of}\;\mathrm{free}\;\mathrm{MTX}\right)}{\mathrm{Total}\;\mathrm{concentration}\;\mathrm{of}\;\mathrm{MTX}}\times 100$$

Then, the drug release kinetics of MTX/FA/GO were also investigated using MATLAB R2023a. The following zero-order (Equation (2)), first-order (Equations (3) and (4)), Korsmeyer–Peppas (Equation (5)), and Higuchi (Equation (6)) models [40] were fitted to the releasing data as they are the most commonly used models in drug release kinetics studies [41].

(2) $$Q=Q_{0}+Kt$$

(3) $$\frac{d\left(100-Q\right)}{dt}=-K\left(100-Q\right)$$

(4) $$Q=100-\left(100-Q_{0}\right)\;e^{-Kt}$$

(5) $$Q=K\;log\;\left(t\right)+Q_{0}$$

(6) $$Q=Q_{0}+K\;t^{0.5}$$

where Q is the cumulative drug release (%), $Q_{0}$ is the initial drug release, K is the rate constant for individual models, and t is time.

The model performance was evaluated using the calculation of R^2^ and root mean square error (RMSE) values (Equations (7) and (8)) [42,43]. The R^2^ value indicates the degree of correlation between the model and the data, while the RMSE measures the average difference between the model’s predictions and the actual values, providing an evaluation of the model’s accuracy in estimating the target value [44].

(7) $$R^{2}=1-\left[\frac{\sum_{i=1}^{m}{\left(y_{i}-{\hat{y}}_{i}\right)}^{2}}{\sum_{i=1}^{m}{\left(y_{i}-\bar{y}\right)}^{2}}\right]$$

(8) $$RMSE=\sqrt{\frac{\sum_{i=1}^{m}{\left(y_{i}-{\hat{y}}_{i}\right)}^{2}}{m}}$$

where $y_{i}$ is the *i^th^* experimental data, ${\hat{y}}_{i}$ is the value calculated by the model for the *i^th^* experimental data, $\bar{y}$ is the mean of all the experimental data, and m is the number of samples.

Finally, the models obtained and their performance were compared with each other, and the best-performing model was illustrated with its 95% prediction bounds to highlight the experimental uncertainties.

### 2.6. Stiffness

The effect of the synthesized GO/MTX formulation on the biomechanical structure of cancer cells was investigated using the acousto-holographic methodology presented by Varol et al. [45,46]. In summary, the modulus of elasticity of the cell was calculated based on the displacement parameter that occurs in the cell membrane after the cells are exposed to acoustic pressure. Within the scope of the experiment, the cells were seeded into the microfluidic chamber. Afterwards, the cells were exposed to acoustic pressure at a frequency of 1 kHz and a voltage of 20 V, and cell stiffness was calculated for the control, MTX-treated, and MTX/FA/GO-treated cell groups.

### 2.7. Cell Culture

The MDA-MB-231 cell line was cultured in DMEM/F-12 cell culture medium containing 10% FBS and 1% pen-strep, respectively, in a CO_2_ incubator at 37 °C. After the cells became confluent, they were washed with PBS (pH: 7.2) and detached from the surface using trypsin-EDTA. Then, the cells were centrifuged at 1000 rpm for 5 min, the supernatant was removed, and the pellet was stained with trypan blue and counted using a Thoma chamber.

### 2.8. Cytotoxicity Experiment

Cytotoxicity analysis was performed to examine the in vitro anticancer activity of the synthesized MTX, GO, and MTX/FA/GO material on a highly aggressive breast cancer cell line, MDA-MB-231, and to find the effective dose range. The anticancer activities of only MTX and only GO were also analyzed to perform a comparative analysis. As the control group, only the cells treated with the cell culture medium were used.

First, MDA-MB-231 cells were seeded into 96-well cell culture plates at 10 × 10^3^ cells per well. After the cells were confluent, they were treated with MTX, GO, and MTX/FA/GO substances in the concentration range of 1–80 µg/mL for 24 h. After incubation, all the supernatant on the cells was aspirated, and 100 µL of XTT solution prepared in the medium containing 0.4 mg/mL concentration of XTT and 7.5 mg/mL PMS was added to each well. After an incubation period of 4 h, absorbance values were measured at 450 nm [47].

## 3. Results and Discussion

### 3.1. Zeta Potential, Size, and Morphology of the GO and Drug Delivery System

**Figure 2:**
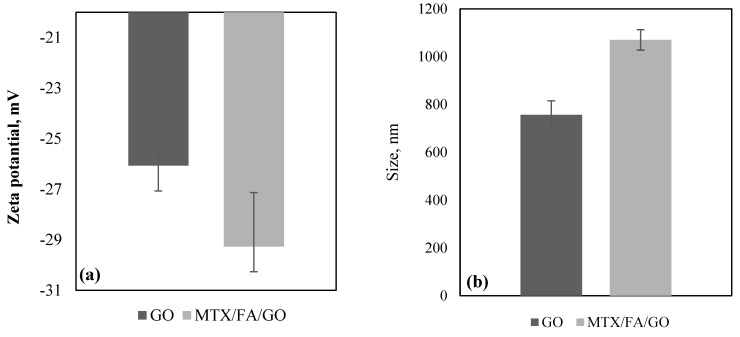
(**a**) Zeta potential (mV) and (**b**) size (nm) of GO and MTX/FA/GO drug delivery system, respectively. GO: graphene oxide; MTX: methotrexate; FA: folic acid.

The zeta potential indicates whether particles are attracted to or repelled by each other. The stability of the particles is dependent on the zeta potential. A larger zeta potential value, whether positive or negative, indicates increased stability and a better likelihood of the particles resisting aggregation. Conversely, a lower zeta potential results in more attraction than repulsion and an irregular distribution [48]. The results of the zeta potential measurement in deionized pure water (pH = 7.0) are shown in Figure 2a. The colloidal stability of chemically transformed graphite, GO (−26 mV), in aqueous medium was observed to have a negative zeta potential. This negative zeta potential is probably due to the presence of –COOH groups on the GO surface [49]. A small decrease in the magnitude of the zeta potential of GO (−26 mV) was seen in MTX/FA/GO (−29 mV); this might indicate the possible formation of amide bonds of MTX and FA with GO. The zeta potentials of GO and MTX/FA/GO may indicate that they can form a stable colloidal structure.

**Figure 3:**
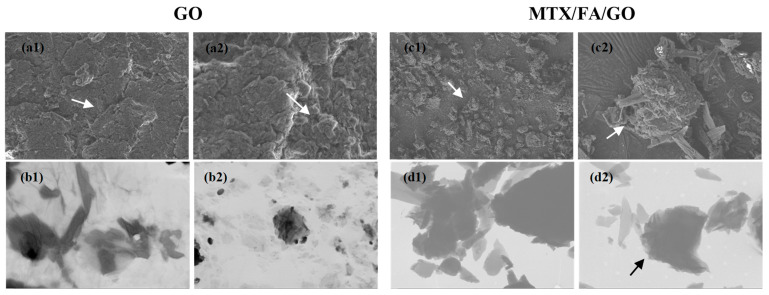
(**a1**,**a2**) SEM images and (**b1**,**b2**) TEM images of GO and (**c1**,**c2**) SEM images and (**d1**,**d2**) TEM images of MTX/FA/GO drug delivery system. GO: Graphene oxide, MTX: Methotraxade, FA: Folic acid, respectively. Scale bar: (**a1**) 2 μm; (**a2**) 1 μm; (**b1**) 200 nm; (**b2**) 1 μm; (**c1**) 10 μm; (**c2**) 2 μm; (**d1**) 400 nm; (**d2**) 1 μm.

Average particle size measurement was performed for GO and GO/MTX/FA. The average particle size was 756 nm for GO. When MTX and FA were connected to GO, the size of the structure increased to 1070 nm. In Table S1, the polydispersity index (PDI) values of GO and MTX/FA/GO are given. The low PDI value is a measure of the size-based heterogeneity of a given sample, with the PDI value for GO measured at 0.28 ± 0.01 and for MTX/FA/GO at 0.35 ± 0.03. This situation, in parallel with the SEM and TEM images (Figure 3), may indicate that the GO structure changes from two-dimensional to three-dimensional with the binding of MTX and FA.

Scanning electron microscopy (SEM) and transmission electron microscopy (TEM) were performed to examine the structure and surface morphology of the GO and MTX/FA/GO drug delivery systems. The SEM images of GO in Figure 3a1,a2 show a characteristic nanosheet structure in which GO layers are stacked on top of each other to form a flake-like two-dimensional structure. This result confirmed that two-dimensional nanosheets of GO could be produced from exfoliation of graphene oxide. Similar types of images have been reported in the literature [4,5,6,7,8,9,10,11,12,13,14,15,16,17,18,19,20,21,22,23,24,25,26,27,28,29,30,31,32,33,34,35,36,37,38,39,40,41,42,43,45,46,47,48,49,50,51,52,53]. The SEM images of MTX/FA/GO in Figure 3c1,c2 show the structures that have formed a three-dimensional shape by creating a raised structure on these flake layers (white arrows). In the TEM images, GO appears in a leaf-shaped and thin form, similar to its two-dimensional structure, while the images of MTX/FA/GO show a three-dimensional form (black arrow) with a higher elevation (Figure 3d1,d2). As seen in Figure 2b, MTX and FA binding to GO significantly increased the particle size from 756 nm to 1070 nm, which is consistent with the morphological images.

**Figure 4:**
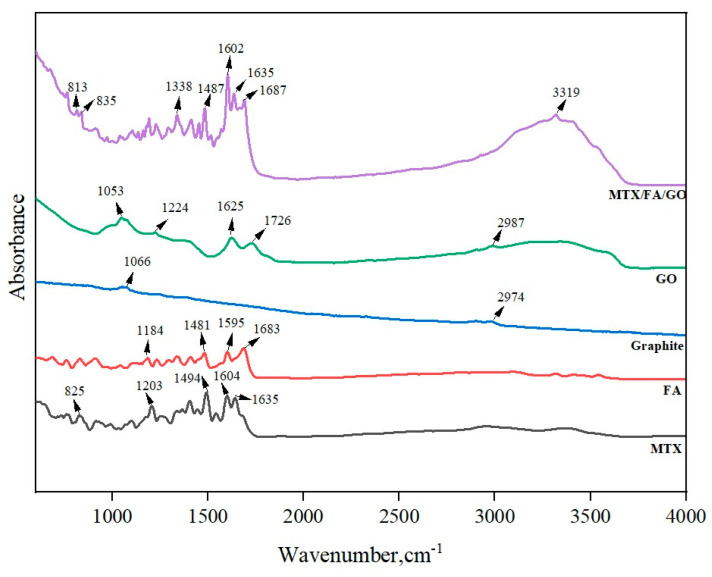
FTIR spectra of pure GO, graphite, FA, MTX, and MTX/FA/GO drug delivery systems. GO: graphene oxide; MTX: methotrexate; FA: folic acid.

Fourier transform infrared spectroscopy (FTIR) is a technique used to analyze the presence of various functional groups of chemical components and synthesized materials. GO, the oxide form of graphite, is expected to contain more oxygen-containing functional groups after the oxidation process. In Figure 3, a very small number of peaks are seen at 2974 and 1066 cm^−1^ for graphite. For GO, the intense and broad peak appearing at a wavelength of 3320 cm^−1^ confirm the presence of the O-H bond (hydroxyl group). In addition, –C=O stretching (–COOH group) is seen at 1726 cm^−1^, and C-O-C stretching (epoxy group) is seen at 1224 and 1053 cm^−1^ wavelengths. The presence of all these carboxylic, hydroxyl, epoxide, and carbonyl groups may indicate that oxygen atoms (O) are bound to GO, and as a result, GO is successfully synthesized [54]. As shown in Figure 4, the peaks at 1625, 1726, and 3320 cm^−1^ confirm the existence of C=C, C=O, and O–H bonds in GO, respectively [51]. The FTIR spectrum of FA shows characteristic peaks at 1683, 1595, and 1481 cm^−1^, corresponding to C=O stretching, aromatic C=C stretching, and the absorption band of the phenyl ring, respectively. The shift of the carboxylic band at 1683 cm^−1^ in FA to 1687 cm^−1^ in MTX/FA/GO may indicate the interaction among the chemical groups of FA with GO. Additionally, an intense band is observed at 1635 cm^−1^, corresponding to amide bond formation between GO, FA, and MTX. In addition, two additional peaks are observed in the MTX/FA/GO spectrum at 813 cm^−1^ and 3319 cm^−1^, corresponding to the primary amine group and -NH groups present in MTX. These data confirm that MTX has been successfully loaded on FA/GO [48].

**Figure 5:**
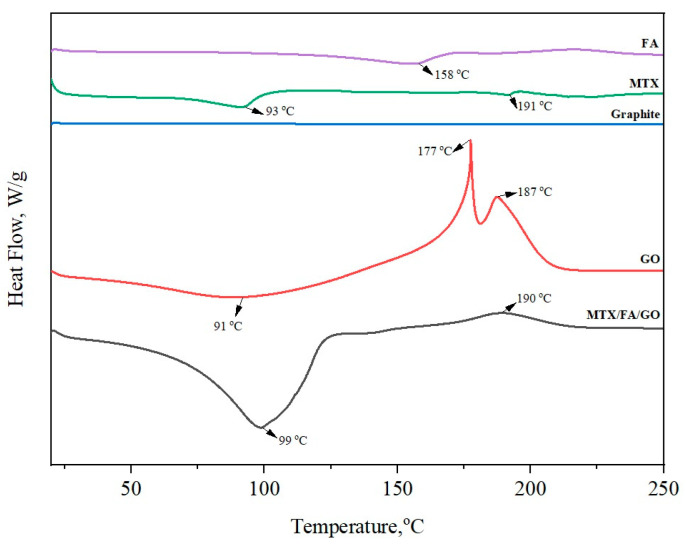
DTG thermograms of pure GO, graphite, FA, MTX, and MTX/FA/GO drug delivery system. GO: graphene oxide; MTX: methotrexate; FA: folic acid.

DSC was used to evaluate the crystallinity, amorphism, and possible physicochemical interactions of the compounds (Figure 5). Graphite powder did not show any decomposition peaks. GO, on the other hand, showed an endothermic peak at 91 °C and an exothermic peak at 177 and 187 °C, similar to a previous study [55]. The decomposition peaks found in GO, unlike in graphite powder, are due to chemical bonds with oxygen. The decomposition peaks of GO at 177 and 187 °C can be attributed to the decomposition of oxygenic groups into CO_2_, resulting from the catalytic dehydration of epoxy (C-O-C), hydroxyl (O-H), and carboxyl (-COOH) bonds [56]. The DSC curve of MTX exhibited an endothermic peak at 93 and 191 °C, while the FA thermogram showed an endothermic peak at 158 °C. Melting peaks of FA and MTX at 158 and 191 °C, respectively, were not observed in the MTX/FA/GO drug delivery system. The absence of phase transitions due to MTX and FA in the DSC analysis may indicate that they bind to GO in a non-crystalline state on the MTX/FA/GO surface [57].

### 3.2. In Vitro Study: Franz Diffusion Drug Delivery Kinetics

The utilization of mathematical models to analyze the release kinetics of pharmaceuticals from different formulations is of utmost importance in comprehending the rate and magnitude of drug release during a certain time period. To this end, zero-order, first-order, Korsmeyer–Peppas, and Higuchi models were fitted to the experimental data in order to estimate the cumulative drug release (%) as a function of time. The model parameters and the models’ performance metrics are given in Table 1.

**Figure 6:**
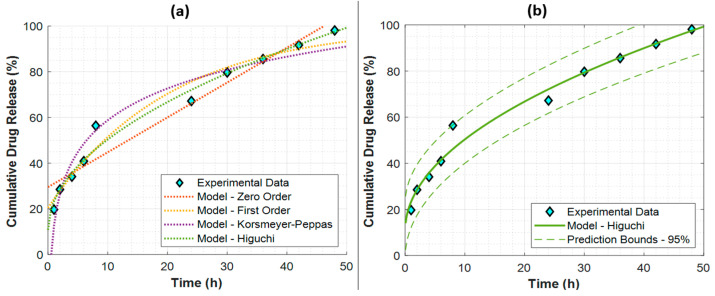
The drug release kinetics of MTX/FA/GO nanomaterials with respect to (**a**) all kinetic models and (**b**) the best-performing kinetic model with its 95% prediction bounds. GO: graphene oxide; MTX: methotrexate; FA: folic acid.

Here, it is clear that the Higuchi model is superior, with the highest R^2^ and lowest RMSE values. All the obtained models are presented with respect to the experimental data in Figure 6a to further validate the remarkable performance of the Higuchi model. The best fit to the Higuchi model of the experimental data also suggests Fickian diffusion for the drug release kinetics of MTX/FA/GO nanomaterials [49].

The best-performing Higuchi model is also plotted with its 95% prediction bounds in Figure 6b. Here, when the MTX/FA/GO release kinetics were investigated, it was observed that the highest MTX release reached 98.1% after 48 h.

### 3.3. Stiffness

**Figure 7:**
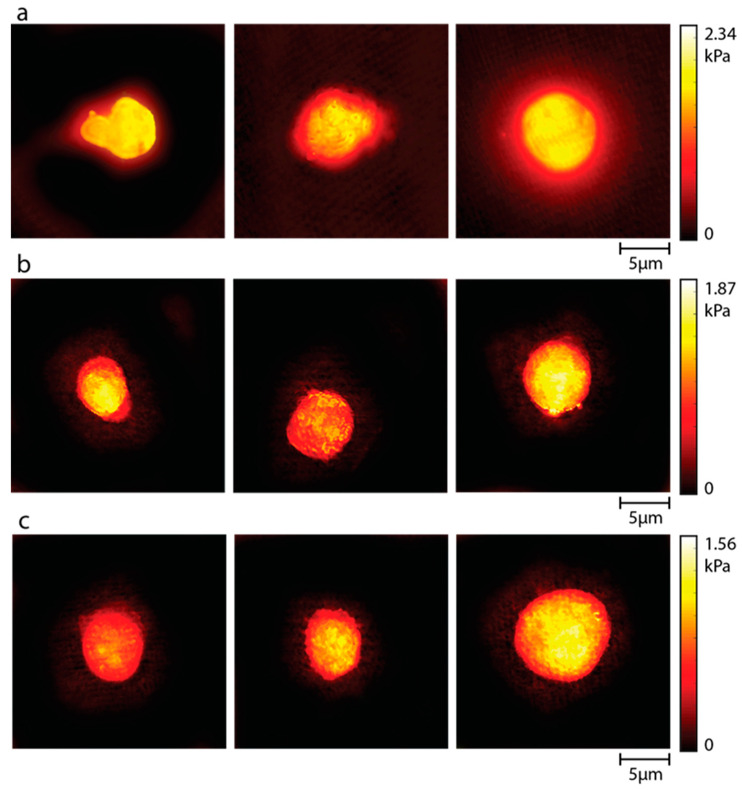
The average cell stiffness distribution of MDA-MB-231 cells: (**a**) control group, (**b**) MTX-treated cells, (**c**) MTX/FA/GO-treated cells. The average stiffness values were found to be 2.34 kPa, 1.87 kPa, and 1.56 kPa, respectively. GO: graphene oxide; MTX: methotrexate; FA: folic acid.

Cell stiffness is one of the basic biomechanical properties of the cell and is associated with the migration, invasion, and metastasis properties of cancer cells. The changes in the cell stiffness of cancer cells after treatment with the produced formulation are shown comparatively in Figure 7. The results show that the stiffness of cancer cells treated with MTX and formulation decreased compared to the control group. The mean cell stiffness was 2.34 kPa for the control group, 1.87 kPa for cells treated with MTX only, and 1.56 kPa for cells treated with the MTX/FA/GO formulation, respectively.

The literature shows that both materials have a unique effect on cells at the single-cell level. The biomechanical properties of the cell are very strong biomarkers that provide information about the cell state. Understanding the effect of an active substance on the biophysical state of the cell is an important step for following cancer progress and drug discovery. Li et al. [58] examined the effect of MTX on cell biomechanical properties and reported that cell stiffness decreased at the single-cell level after exposure to the drug. These changes in the viscoelastic properties of the cells treated with MTX were associated with significant morphological changes, such as vanished filamentous structures and rounded cells. In addition, in the studies of Ghorbani et al. [59], the effects of GO sheets on the biomechanical properties of MCF-7 and MDA-MB-231 cells, which are breast cancer cell lines, were investigated. The results show that there was a significant decrease in the cell stiffness of cells treated with GO. They associated this with changes in the organization of actin filaments after treatment. Zhu et al. [60] reported that the permeability of the membranes of cancer cells increased after treatment with GO, making them more suitable for drug delivery. In our study, the decrease in stiffness of cells treated with the MTX/GO complex compared to the control group and groups treated with MTX alone may be associated with altered membrane permeability, particularly due to GO. In addition, cytotoxicity analysis results, in parallel with the stiffness results, showed that the co-application of MTX/FA/GO complex had a 35% more cytotoxic effect on cancer cells at a concentration of 80 mg/mL than MTX alone (Figure 4). This indicates that the targeted MTX/FA/GO complex containing FA, with weakened cell membrane stiffness, increases cytotoxicity by causing greater cell penetration. Functionalized GO has been reported to enhance anticancer activity by using receptor-mediated endocytosis to deliver anticancer drugs to cells in an efficient and focused manner [61].

### 3.4. Cell Culture and Cytotoxicity Analysis

**Figure 8:**
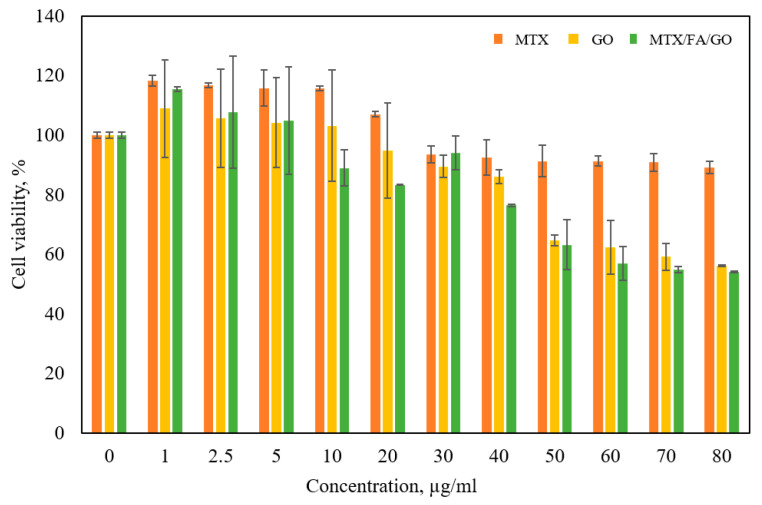
The effect of MTX, GO, and MTX/FA/GO formulations on the viability of MDA-MB-231 cells at different concentrations. GO: graphene oxide; MTX: methotrexate; FA: folic acid.

Figure 8 indicates that MTX/FA/GO begins to show significant cytotoxicity on cancer cells, especially at a concentration of 10 µg/mL. Cell viability was found to be 89.01%, 83.36%, 94%, 76.42%, 63.22%, 56.90%, 54.89%, and 54.14% for 10 µg/mL, 20 µg/mL, 30 µg/mL, 40 µg/mL, 50 µg/mL, 60 µg/mL, 70 µg/mL, and 80 µg/mL concentration values, respectively. As shown in the graph, the MTX/FA/GO material obtained by synthesizing GO together with MTX showed more significant anticancer activity on cancer cells compared to MTX and GO. This may be associated with the synergistic effect of GO and MTX. In addition, considering that folate receptors are overexpressed in cancer cells [62], the inclusion of FA in the MTX/FA/GO micro-material ensures that the structure becomes target-specific. In addition, it has been revealed by various sources [63] that FA may have a curative effect on breast cancer, depending on the concentration.

The folate receptor (FR), which is present on the cell surface, is used in the receptor-facilitated endocytic route to transport FA into cells. The most extensively expressed FR isoform among the FR receptors is FRα, which is overexpressed in a number of cancerous tissues, including lung, ovarian, cervical, kidney, and breast tumors [64]. To ensure better drug retention by cancer tissue, a variety of folic acid-conjugated nanocarriers have been produced [65,66]. Watanabe et al. (2012), for instance, showed how to use folate-polymer-coated liposomes for the targeted delivery of DOX [65]. They showed that due to increased cellular internalization than human lung adenocarcinoma (A549) cells, DOX-loaded FA-conjugated liposomes demonstrated twofold higher cytotoxicity against FR-overexpressing human nasopharyngeal carcinoma (KB) cells. In the light of this information, the MTX/FA/GO component may be an effective option for increasing the efficacy of the MTX anticancer drug and gaining a target-specific structure.

Giusto et al. [67] showed that GO@PEG material inhibits migration on MDA-MB-231 cells and can be used as a promising anticancer material. De et al. [68] tested the anticancer activities of curcumin-GO complexes on MCF-7 and MDA-MB-468 breast cancer cell lines. It has been shown that these complexes increase anticancer activity compared to GO or Cu alone. Xu et al. [69] loaded doxorubicin (DOX) into GO hybridized nanogels and tested its anticancer activity on A549 cells. As a result, they showed that the intracellular delivery of DOX drug was increased by GO nanogels. Marrella et al. [70] showed that migration and invasion of breast cancer cells are inhibited after treatment with GO and that tumor growth and metastasis are also inhibited by inhibiting mitochondrial respiration. Other studies have also reported that GO functionalized with MTX has an increased cytotoxic effect on cancer cells [23,71,72]. It has also been shown by stiffness analysis that the combined use of MTX with FA and GO increases the cytotoxic effect by changing cell mechanics. The cell wall was weakened more in MTX/FA/GO application compared to free MTX application, which increased drug penetration. The conclusion that the GO + MTX combination shows a predominant anticancer activity compared to GO or MTX alone is supported by the literature, considering the potential of GO to increase anticancer activity.

## 4. Conclusions

The nanosheet structure of GO was verified by SEM and TEM images, and it was demonstrated that the binding of MTX/FA to GO transformed two-dimensional GO into a three-dimensional structure. The successful synthesis of GO was confirmed by FTIR and DSC graphs, which showed that oxygen molecules were attached to GO through the production of carboxylic, hydroxyl, epoxide, and carbonyl groups as a result of graphite’s oxidation. These analyses also demonstrated the physicochemical binding of MTX and FA to the GO structure. The cumulative release results showed that nearly 100% of MTX/FA/GO was released from the system after 48 h. Using Franz diffusion analysis, it was observed that MTX diffused from the MTX/FA/GO drug release system according to the Higuchi model, which suggests Fickian diffusion. The combined use of the MTX/FA/GO drug delivery system had a higher cytotoxic effect on MDA-MB-231 cells than free MTX. Stiffness analyses showed that the addition of FA to this release system directs the system to target cells, facilitating the entry of the drug into the cell. As a result of stiffness analyses, the stiffness values of the control cell group, free MTX, and MTX/FA/GO applied cells were found to be 2.34 kPa, 1.87 kPa, and 1.56 kPa, respectively. With these results, it was seen that MTX/FA/GO weakened the cells.

When MTX/FA/GO nanomaterials were analyzed, 96% MTX release was revealed in the release kinetics study. It was observed that MTX/FA/GO formulation may be more suitable as a drug delivery system. With this study, we demonstrated that MTX-linked GO functionalized with folic acid (FA) could be an alternative nanocarrier for the delivery of anti-tumor drugs. Cell stiffness analysis also showed that GO was efficiently functionalized with FA. It was observed that conjugation of FA, where the drug can accumulate more on the GO surface area, can increase the functionality of MTX on the GO surface area. In vivo studies should be carried out first to determine whether it will be useful in targeted anticancer therapies and chemodynamic therapy (CDT).

## Figures and Tables

**Table 1:** Model parameters and performance metrics for the drug release kinetic models of MTX/FA/GO nanomaterials.

Model	Parameters		Metrics	
	K (%)	Q_0_ (%)	R^2^	RMSE (%)
Zero-Order	1.5259 1/h	29.5187	0.9425	7.2031
First-Order	4.9400 1/h	20.4237	0.9677	5.3938
Korsmeyer–Peppas	20.0631	12.5957	0.9554	6.3418
Higuchi	12.5140 1/h ^0.5^	10.7882	0.9799	4.2599

## Data Availability

The data presented in this study are available on request from the corresponding author. The data are not publicly available due to ongoing researches.

## Supplementary Materials

The following supporting information can be downloaded at: https://www.mdpi.com/article/10.3390/pharmaceutics16060837/s1, Table S1. Z-average size (nm) and PDI result of GO, and MTX/FA/GO drug delivery system. GO: Graphene oxide, MTX: Methotraxade, FA: Folic acid, PDI: Polydispersity index, respectively.
