# Supplementary material for: Development of Graphene Oxide-Based Anticancer Drug Combination Functionalized with Folic Acid as Nanocarrier for Targeted Delivery of Methotrexate
Source: Pharmaceutics. 2024 Jun 20;16(6):837. doi: 10.3390/pharmaceutics16060837 (PMC11207743; doi:10.3390/pharmaceutics16060837)
Supplement: Supplementary file 1 [file pharmaceutics-16-00837-s001.zip › pharmaceutics-3039625-supplementary.pdf]

Table S1. Z-average size (nm) and PDI result of GO, and MTX/FA/GO drug delivery system. GO: Graphene oxide, MTX: Methotraxade, FA: Folic acid, PDI: Polydispersity index, respectively.

Samples	Z-average size (nm)	PDI
GO	756,66±8,56	0,28±0,01
MTX/FA/GO	1070±10,41	0,35±0,03
